# Family intervention therapy in alcohol dependence syndrome: One-year follow-up study

**DOI:** 10.4103/0019-5545.37322

**Published:** 2007

**Authors:** P. N. Suresh Kumar, Biju Thomas

**Affiliations:** Assistant professor of Psychiatry, Govt. Medical College, Trivandrum, Kerala, India; * Consultant Psychiatrist, ICCONS, Shornur, Palaghat, Kerala, India

**Keywords:** Alcohol dependence, family intervention therapy, pharmacotherapy, psychotherapy

## Abstract

**Background:**

Among the various treatment modalities, family intervention is the most notable current advance in the area of psychosocial treatment of alcoholism.

**Aim:**

To assess the impact of family intervention therapy as an adjuvant to pharmacotherapy in alcohol-dependent subjects in a case-control study design.

**Materials and Methods:**

Thirty patients who satisfied DSM-IV Criteria for alcohol dependence syndrome were given the right package of family intervention therapy. Thirty age-, sex- and ‘marital status’-matched patients who satisfied the same diagnostic criteria were given only brief supportive psychotherapy. Both groups were assessed at base line, six months and at one year using Michigan Alcohol Screening Test, Motivation Scale, Rotter's Locus of Control, Family Intervention Pattern Scale and Presumptive Stressful Events Scale. Primary efficacy variable was cumulative abstinence duration, and secondary efficacy variables were relapse rate and time to first drink.

**Results:**

Family intervention therapy significantly reduced the severity of alcohol intake, improved the motivation to stop alcohol and changed the locus of control from external to internal in the study group. Control group experienced more severe stressful life events than the study group during the follow-up periods. Drop-out rate was comparable in both groups.

**Conclusion:**

Combining pharmacological treatment with appropriate psychosocial therapies focusing on the specific problem of the patient provides better outcome than either of these therapies given alone.

Alcohol dependence is a complex behavior with far-reaching harmful effects on the family, work, society, as well as on the physical and mental health of the individual. Epidemiological studies conducted in India showed that 20-30% of our population is using alcohol at a harmful level.[1–6]

Heavy alcohol consumption exerts a deleterious effect on the family.[7] The extent of the negative impact varies among family members and from family to family. It often results in serious emotional and medical problems. Family intervention treatment in the field of alcoholism is a relatively new phenomenon. Family members' negative responses to the alcoholic's behavior usually reinforce the individual's alienation and dependency resulting from alcoholism.[8]

An alcohol-dependent person seeks professional help mostly persuaded by his wife, family members, neighbors, co-workers, employer, etc. Need for immediate care may be due to a threat of divorce, dismissal from job, serious injury due to fall, aborted marriage proposal to his ward, health hazards, etc. Many studies conducted in the field of alcoholism have concluded that better outcome is possible when alcohol-dependent persons receive nonpharmacological therapy along with pharmacological treatment.[9–14] However, most of these studies were confined to selective psychotherapy techniques, leaving the comprehensive psychosocial treatment to be an unexplored area.

Among the various treatment modalities, family intervention is the most notable current advance in the area of psychosocial treatment of alcoholism. Family intervention is a method of understanding and encouraging the role of family, and it imparts positive effect in decreasing alcohol consumption.[14] The ingredients of this mode of therapy include (1) building up an alliance with relatives (2) reducing adverse family atmosphere (3) enhancing problem-solving capacity of family members (4) decreasing of anguish and repentance (5) maintaining reasonable expectations for patient performance (6) achieving changes in family members' behavior and belief system.

A kind of a package consists of psychoeducation, family therapy, cognitive behavior therapy and behavioral counseling of spouses. Two broad aims of family intervention packages are (a) reduction of tension in the family atmosphere and (b) improvement of the social functioning of the patient.

Many alcoholics have extensive marital and family problems, and hence positive marital and family adjustment is associated with better outcome. It has been reported that even at the onset of recovery from alcohol dependence, marital and family conflicts may often precipitate and lead to relapses in abstinent alcoholics.[14] Till date, there are no studies reported from India assessing the efficiency of combined family intervention therapy package with pharmacotherapy.

## OBJECTIVES

- Assessment of abstinence rate, severity of alcoholism, motivation for change, locus of control, family interaction pattern and life events preceding relapse.
- Assessment of impact of family intervention therapy as an adjuvant to pharmacotherapy in the outcome of alcoholism.

## MATERIALS AND METHODS

This study was conducted at the De-addiction Unit of Institute of Mental Health and Neurosciences, Kozhikode. Consecutive patients satisfying DSM-IV criteria[15] for alcohol dependence were selected for this study. Only male inpatients in the age group of 15-45 years during the detoxification period were selected for the study after getting informed consent. Those exhibiting evidence of severe physical disorder or any other axis-1 disorder were excluded from the study.

The study group consisted of 35 inpatients after 10 days of inpatient treatment. The control group consisted of 35 patients unwilling to participate in the family intervention therapy. Control group was individually matched with the study group on variables such as age, sex and marital status. Both groups were given disulfiram treatment as outpatients during the follow-up period.

### Clinical assessments

The psycho-socio-demographic profile and the details regarding the pattern of alcohol use, abstinence and past treatment were documented in a specially designed pro forma. Severity of alcoholism, motivation, locus of control, family intervention pattern and life events were assessed using the respective scales. As far as possible, information was collected with the help of relatives, co-workers and friends.

Patients in the study groups were given family intervention therapy consisting of psychoeducation, counseling, group therapy, marital therapy, family therapy and behavioral counseling for spouses. The right package of therapy was selected for the study group. Patients and their close relatives were included, and appropriate therapy was given as outpatients. Ten to 20 sessions, on an average, were conducted for a period of 45 minutes on a monthly basis. Control group was given only pharmacotherapy with a brief problem-focused supportive psychotherapy. Patients in both groups were followed up after six months and at one year with the assessment using following tools (a) Michigan alcohol screening test (MAST) devised by Selzer, 1971[16]; (b) Motivation Scale by Teresa and Nagalakshmi, 1994[17]; (c) Rotter's Locus of Control by Kumar and Srivastava, 1985[18]; (d) Family Intervention Pattern Scale (FIPS)[19] and (e) Presumptive Stressful Life Events Scale (PSLES).[20]

### Cumulative abstinence duration (CAD)

This primary efficacy variable was defined as the total number of days of abstinence and was calculated as the sum of only periods of complete abstinence. If any relapse was recorded at a specific visit, the total period from the previous visit was considered as relapse, although this method will overestimate the length of relapse period. Any alcohol consumption was considered as a relapse, even if alcohol consumption was limited. Missing data was designated as “not abstinent.” The relapse rate based on the reported alcohol consumption was determined at each visit. The patients were categorized as being in “abstinent,” “relapse” or “not abstinent.”

Secondary efficacy variables include time to first relapse and the proportion of patients remaining abstinent during the entire study period.

### Data analysis

The data was analyzed using SPSS software system. Parametric and nonparametric variables were compared using Chi-square (with Yates's correction) and Student's ‘*t*’ test, respectively. Comparison of proportion was done by Fisher's Exact Probability test. Repeated measurements were compared between the two groups using 2-way ANOVA. Time to first relapse was compared between the two groups by survival analysis by Lee-Desu test.

## RESULTS

Out of 35 the patients selected for family intervention therapy, 30 completed one-year follow-up in the study group and in the control group. The reasons for dropout were comparable in both the groups. Different socio-demographic variables were also comparable in both groups [Table 1].

**Table 1:** Comparison of socio-demographic variables between study group and control group

	Study group N = 30	Control group N = 30	Significance (*P*)
Mean age	40.3 + 12.8	41.3 + 11.4	NS
Domicile			
Rural	20 (66.7)	19 (63.3)	NS
Urban	10 (33.3)	11 (36.7)	
Marital status			
Married	28 (93.3)	28 (93.3)	NS
Religion			
Hindu	16 (53.3)	11 (36.7)	0.05
Muslim	2 (6.7)	1 (3.3)	
Christian	12 (40.0)	18 (60.0)	
Mean monthly income (Rupees)	4866 + 129.6	4800 + 120.4	NS
Mean education (Years)	11.6 + 1.5	10.4 + 2.4	NS
Occupation			
Business	12 (40.0)	13 (43.3)	NS
Manual labor	6 (20.0)	5 (16.7)	
Farmer	3 (10.0)	6 (20.0)	
Professional	3 (10.0)	2 (6.67)	
Unemployed	1 (3.3)	0 (0.0)	
Others	5 (16.7)	4 (13.3)	

Nearly half of the patients in both groups expressed unsatisfactory marital adjustment (56.7% in the study group *vs*. 66.7% in the control group). Mean duration of drinking (6.8 + 2.69 *vs*. 6.68 + 3.8 years) was comparable in both groups. As many as 33.3% of the study group had previous treatment compared to 63.3% of the control group. Cumulative abstinence duration and relapse rate were significantly longer in the study group. Also, the time to first relapse was significantly increased in the study group (median = 55.5 days) in comparison with the control group (median = 15 days) [Table 2].

**Table 2:** Comparison of social and clinical variables related to drinking behavior

	Study group N = 30	Control group N = 30	Significance (*P*)
Marital adjustment			
Satisfactory	10 (33.3)	8 (26.7)	NS
Unsatisfactory	17 (56.7)	20 (66.7)	
Not applicable	3 (10.0)	2 (6.7)	
Sexual adjustment			
Satisfactory	20 (66.7)	17 (56.7)	NS
Unsatisfactory	7 (23.3)	11 (36.7)	
Not applicable	3 (10.0)	2 (6.7)	
Mean duration of drinking (year)	6.8 + 2.69	6.68 + 3.8	NS
Prior treatment for alcoholism	10 (33.3)	19 (63.3)	<0.05
Family history of alcoholism	13 (43.3)	15 (50)	NS
Initiating factor			
Peer pressure	25 (83.3)	24 (80)	NS
Family drinking	19 (63.3)	13 (43.3)	
Stressors	14 (46.7)	13 (43.3)	
Maintaining factor			
Craving	16 (53.3)	15 (50.0)	NS
Peer pressure	26 (20.0)	5 (16.7)	
Withdrawal symptoms	5 (16.7)	6 (20.0)	
Stressors	3 (10.0)	4 (13.3)	
Cumulative abstinence duration (days)	212.79 + 19.22	174.93 + 21.88	<0.05
Time to first relapse in days (median)	55.5	15	<0.05
Relapse rate			
Complete abstinence	15(50)	8(26.6)	<0.05

Table 3 shows significant reduction in severity of alcohol intake at the sixth month and at one-year follow-up in the study group by the MAST score. It also shows that patients who received family intervention therapy had attained significantly more internal locus of control at six months and at one-year follow-up. The study group patients were found to be more motivated for a change at the time of each of the two follow-ups. They showed higher self-esteem, better internal locus of control, better growth motivation, higher religious attitude and self-criticality than did the control group at each follow-up [Table 3].

**Table 3:** Comparison of the two groups based on specific variables under study

Score of various scales	Period	Study group Mean ± SE	Control group Mean ± SE	*P*-value
Mast	Initial	20.8 ± 1.97	28.3 ± 1.31	−0.03
	6^th^ month	1.0 ± 0.49	19.3 ± 2.5	−6.84**
	1 year	2.8 ± 1.00	20.9 ± 2.64	−5.80**
Locus of control	Initial	17.2 ± 0.62	16.1 ± 0.62	1.29
	6^th^ month	8.5 ± 0.78	14.1 ± 2.57	−4.19**
	1 year	11.0 ± 0.95	15.0 ± 2.74	−2.57**
Motivation for change	Initial	192.8 ± 5.84	201.2 ± 5.46	1.96
	6^th^ month	245.2 ± 4.16	195.4 ± 9.60	4.64**
	1 year	231.0 ± 4.78	176.6 ± 10.89	4.38**
FIPS	Initial	237.6 ± 4.76	244.3 ± 3.54	−1.80
	6^th^ month	142.9 ± 5.40	202.6 ± 9.21	−5.10**
	1 year	154.1 ± 6.48	211.21 ± 10.89	−4.34**
PSLE	Initial	24.3 ± 1.15	24.0 ± 1.04	0.15
	6^th^ month	5.4 ± 0.55	6.8 ± 0.56	−1.69
	1 year	5.4 ± 0.69	7.8 ± 0.67	−2.39*

* *P* < 0.05;

** *P* < 0.01

Comparison of mean total score of FIPS of both groups at the sixth month and at one-year follow-up showed significant improvement in the study group. Comparison of subgroups, namely, reinforcement, social support, role, communication, cohesiveness and leadership, showed significant improvement at each follow-up in the study group. Also, at the one-year follow-up, life event score was significantly less in the study group.

## DISCUSSION

Alcohol dependence is a serious social, psychological andmedical problem in many parts of the world. The rapid growth of this enigmatic problem across the world, as well as the heterogeneity of the clients, involved the ramifications of the medical and social consequences and the development of multimodel treatment facilities - all these make definitions of an ideal treatment approach difficult.

Present study showed higher motivation for change, more internal locus of control and improved family interaction pattern in patients who received a combination of pharmacotherapy and the right package of family intervention therapy. Analysis of primary efficacy variables also showed significantly higher cumulative abstinence, reduced relapse and increased time to relapse in patients who received family intervention in addition to pharmacotherapy. Advantage of combining psychosocial management in the treatment of alcoholism is in line with the previous reports.[10–13] However, most of these studies have used selective psychosocial management techniques, leaving the comprehensive psychosocial treatment to be an unexplored area.

Engaging the family of the alcohol dependents is definitely helpful in providing support for the patients and in helping them to remain under treatment. Family intervention is also helpful to prevent problems of the spouse or children of alcoholics.[21] At the beginning of the study, most of the families were characterized by poor communication pattern, lack of mutual warmth and support, poor role functioning, lack of leadership and spouse abusing. Spouses of alcoholics expressed greater dissatisfaction in all areas of family functioning. After family intervention therapy, these families expressed greater satisfaction in family functioning, such as free and open communication, mutual warmth and support, becoming ideal role models, evincing good leadership, cohesiveness and sharing of responsibilities. Communication and the learning of problem-solving skills provided the couple with additional behavioral skills to cope up with relapse episodes. This extended treatment package also taught the subjects the skill to maintain abstinence from alcohol, to a greater extent compared to the restricted brief psychotherapy group. Previous studies have demonstrated better outcome in terms of treatment compliance, subjects' ability to cope with drinking, marital stability and subjective well-being when individual alcoholism treatment was combined with marital or family therapy.[13]

In the beginning of this study, both groups of patients scored low on all aspects of motivation except religious attitudes. At follow-up, patients who received family intervention therapy showed higher motivation for change than did the control group. Motivation to stop alcohol is a good factor and it facilitates positive change in the individual. After therapy, these patients also showed significantly higher self-esteem than did the control group.

At the beginning of therapy, the locus of control in both groups was external. Family intervention therapy shifted the locus of control orientation from external to internal in the study group but not in the control group. Changing the locus of control from external to internal could bring about a positive change in the motivational status of alcohol dependents. This implied that patients who received family intervention had gained more control over alcohol and became more responsible for their behavior. Internal locus of control has been proved as a good prognostic factor and facilitated better outcomes in earlier studies.[22]

Understanding the mechanism behind successful outcome in the treatment of alcoholism is still incomplete. Desai *et al*[23] have found that duration of dependence and the number of treatment-related abstinences are the best predictors of successful therapy. In this study, the duration of dependence was comparable in both groups. Same time, the control group had more number of unsuccessful treatments in the past. Moreover, the refusal of the control group for family intervention therapy indirectly denotes poor motivation for change in that group. Apart from willingness for family intervention therapy, the study group also showed greater interest in regular follow-up and in bringing their family members for therapy. The relapsed patients (especially those who had received brief supportive psychotherapy) showed complete disinterest in psychotherapy, and their attitude towards psychotherapy was negative. Majority opined that it was just wasting their valuable time. Perhaps, the genuine interest to accept the therapy is the most crucial indicator in predicting success of treatment.

The drop-out rate in the study and control group (14.3%) was comparable. This is much lesser than the previously reported rates. Western studies have reported 30-35% drop-out rate,[24,25] and studies from India showed 32-50% dropout in the treatment of alcohol dependence.[23,26,27] Probably, involvement of spouse or any other family member in both groups might have influenced the lower dropout in the present study.

In this study, control group patients experienced more severe stressful life events than did the study group during the follow-up periods. Previous studies have also shown an elevated relapse rate associated with acute severe stressors and highly threatening chronic difficulties.[28]

Some of the methodological limitations of this study need to be considered before concluding. The sample size was small to detect minor differences in the outcome between the two groups. Structured diagnostic interview schedule would have given better clarity in the diagnosis, especially with axis-II comorbidity. The therapist who assessed the progress of therapy was not blind to the treatment provided. Future studies with larger sample size, randomization and with longer duration of follow-ups would provide more information on the efficacy of family intervention therapy in the management of alcohol dependence.

## CONCLUSION

The present study suggests that comprehensive multimodal patient-friendly treatments are more effective than any single approach in the management of alcohol dependence. Patients' motivation to accept the mode of treatment is a crucial factor in the success of therapy. Many of the alcohol-dependent patients have impaired marital/family functioning. Hence combining pharmacological treatment with appropriate psychosocial therapies focusing on the specific problem of the patient may provide better outcome than either of these therapies given alone.
